# Fetoplacental transmission and placental response to SARS-CoV-2: Evidence from the literature

**DOI:** 10.3389/fmed.2022.962937

**Published:** 2022-08-16

**Authors:** Henry C. Ezechukwu, Jiahua Shi, Muinah A. Fowora, Cornelius A. Diya, Faiz Elfaki, Oyelola A. Adegboye

**Affiliations:** ^1^Department of Medical Biochemistry, EKO University of Medicine and Health Sciences, Lagos, Nigeria; ^2^School of Human Sciences, University of Western Australia, Perth, WA, Australia; ^3^School of Medical, Indigenous and Health Sciences, University of Wollongong, Wollongong, NSW, Australia; ^4^Illawarra Health and Medical Research Institute, University of Wollongong, Wollongong, NSW, Australia; ^5^Department of Molecular Biology and Biotechnology, Nigerian Institute of Medical Research, Lagos, Nigeria; ^6^Department of Mathematics, Physics and Statistics, College of Arts and Sciences, Qatar University, Doha, Qatar; ^7^Public Health and Tropical Medicine, College of Public Health, Medical and Veterinary Sciences, James Cook University, Townsville, QLD, Australia; ^8^Australian Institute of Tropical Health and Medicine, James Cook University, Townsville, QLD, Australia

**Keywords:** SARS-CoV-2, placenta, vertical transmission, COVID-19, maternal-child

## Abstract

Severe acute respiratory syndrome coronavirus 2 (SARS-CoV-2) is a dreadful novel coronavirus with global health concerns among pregnant women. To date, the vertical transmission of SARS-CoV-2 during pregnancy remains controversial. We briefly report recent findings of placental response to SARS-CoV-2 infection and updates on vertical transmission. We systematically searched PubMed and Google Scholar databases according to PRISMA guidelines for studies reporting the effects of SARS-CoV-2 infection on the placenta and possibility of vertical transmission. We identified 45 studies reporting 1,280 human placentas that were analyzed by molecular pathology methods and 11,112 placenta-derived cells from a publicly available database that was analyzed using bioinformatics tools. The main finding of this study is that the SARS-CoV-2 canonical entry receptors (ACE2 and TMPRSS2) are abundantly expressed on the placenta during the first trimester, and this expression diminishes across gestational age. Out of 45 eligible studies identified, 24 (53.34%) showed no evidence of vertical transmission, 15 (33.33%) supported the hypothesis of very rare, low possibility of vertical transmission and 6 (13.33%) were indecisive and had no comment on vertical transmission. Furthermore, 433 placentas from 12 studies were also identified for placental pathology investigation. There was evidence of at least one form of maternal vascular malperfusion (MVM), 57/433 (13.1%), fetal vascular malperfusion (FVM), 81/433 (18.7%) and placental inflammation with excessive infiltration of CD3+ CD8+ lymphocytes, CD68+ macrophages and CD20+ lymphocytes in most of the eligible studies. Decidual vasculopathy (3.2%), infarction (3.2%), chronic histiocytic intervillositis (6.0%), thrombi vasculopathy (5.1%) were also observed in most of the MVM and FVM reported cases. The results indicated that SARS-CoV-2 induces placenta inflammation, and placenta susceptibility to SARS-CoV-2 decreases across the pregnancy window. Thus, SARS-CoV-2 infection in early pregnancy may adversely affect the developing fetus.

## Introduction

The first case of severe acute respiratory syndrome coronavirus 2 (SARS-CoV-2) infection was reported in Wuhan, China in December 2019, and since March 2020, the World Health Organization declared COVID-19 as a global pandemic (1, 2). Numerous studies have highlighted SARS-CoV-2 infection during pregnancy outcomes such as miscarriages and preterm birth (3–7).

The concern about the possibility of fetoplacental transmission remains an open question. There is controversy about this topic in literature. To begin with, vertical transmission is simply the possible transmission of an infectious pathogen from the maternal side to the developing fetus during the antepartum and intrapartum period, or to the neonate during postpartum via the placenta, *in utero*, body fluid contact during delivery or through breastfeeding (8). The placenta is an essential organ that provides protection and nutrients for the developing fetus, and it happens to be a potent target for viral infection during pregnancy. For example, several studies have also shown that Zika virus infected placenta during pregnancy can induce fetoplacental transmission, which is associated with fetal demise (9–11). In the first quarter of 2021, the World Health Organization released set of criteria for timing of possible vertical transmission of SARS-CoV-2 from mother to child. These criteria were based on three fundamental elements: time of maternal infection, test to evaluate *in utero* and intrapartum exposure and finally test to identify later exposure after neonatal birth (12). Several literature as discussed in this review supports such possibility. One of the few studies that uses these criteria reported positive cases relating to intrauterine transmission of SARS-CoV-2 in neonates (13). Certain respiratory virus have also been seen in vertical transmission to the offspring during pregnancy [see ref (14)], though whether this is a local or systemic effect, still requires further investigation.

To date, findings on fetoplacental transmission of SARS-CoV-2 remain controversial and inconsistent. Therefore, this study summarizes evidence from available literature on the placenta response to SARS-CoV-2 infection and fetoplacental transmission. Our finding will add to the knowledge that SARS-CoV-2 infection might have an adverse effect on the placenta in the first trimester and that more research is required to ascertain the possibility of the occurrence of fetoplacental transmission.

## Materials and methods

### Search strategy

This systematic review was conducted according to the Preferred Reporting Items for Systematic Reviews and Meta-Analyses (PRISMA) guidelines to identify relevant literature (15). The search mainly focused on the mapping of existing literature in placenta response to SARS-CoV-2 infection. We conducted a literature search on articles published from 1st March 2020 to 14th April 2022 on PubMed and Google Scholar databases using Boolean search tools on the following keywords: (((”SARS-CoV-2”) AND (”Placenta”)) AND (”COVID-19”)). Article titles containing the keywords were identified on PUBMED and Google Scholar.

### Study selection and criteria

**FIGURE 1:**
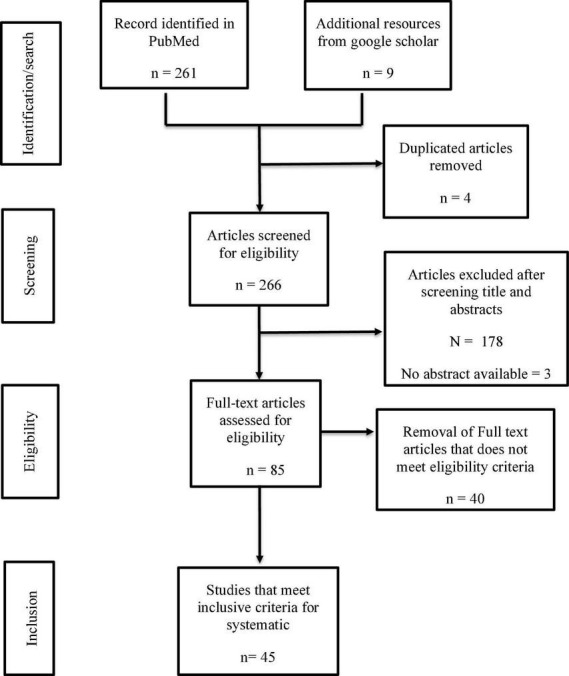
PRISMA flow chart for study selection, eligibility and inclusion.

Using PRISMA format, the results from the database were exported into a CSV file, and initial screening was performed by reading through the titles and abstracts of all the identified studies. Duplicates and studies not meeting the inclusion criteria were excluded. See Figure 1 for details of data search results.

#### Inclusion criteria

Articles were selected and assessed only if they met the following criteria: (1) studies reporting placenta response to SARS-CoV-2 infection. (2) Studies reporting the immunopathological status of the placenta in COVID-19 patients. (3) Studies on whether there is vertical transmission of SARS-CoV-2 infection during pregnancy. (4) Studies must be a research article published in English and indexed in MEDLINE.

#### Exclusion criteria

Studies excluded in this study were narrative literature reviews, meta-analysis reviews, systematic reviews, letters to the editor, abstracts without full text, clinical trials, observational studies, conference proceedings, and studies published before March 2020.

### Data collection process and data items

Data from the full text was collected using an excel spreadsheet. Repeated assessment was performed independently to confirm the accuracy of the extracted data. All discrepancies and disagreements were resolved by consensus. OA and HE review each selected study independently according to the selection criteria until all discrepancies were resolved. JS also review the selection steps at a later time to minimize publication bias.

#### Data extraction, strategy for data synthesis, risk of bias assessment and data analysis

The following parameters were collected: authors name, publication year, number of placenta, SARS-CoV-2 entry receptors, method of SARS-CoV-2 entry receptor detection [PCR, immunohistochemistry (IHC), *in situ* hybridization (ISH)], time (or stage) of pregnancy, and pathological features of SARS-CoV-2 infected placenta. These pathological features include maternal vascular malperfusion (MVM), fetal vascular malperfusion (FVM), status on vertical transmission, and placenta inflammatory response such as Hofbauer cells infiltration and cytokine secretion. The data synthesis was based on the inclusion criteria, which includes a narrative synthesis of the evidence and summary tables showing the findings. We assessed each eligible study for risk of bias based on: (1) randomization of sample collection and selection, (2) homogeneity of the samples, (3) method of detection, (4) incomplete or omission on data outcome, (5) selective reporting of only statistically significant data (16) (Table 2). We presented the risk of bias as follows: High (+), low (–), and unclear (?).

Using fixed and random effects models, we presented the prevalence of maternal-fetal transmission from each study and pooled estimates with associated 95% confidence intervals (CIs). We used the *I^2^ statistic* to assess the heterogeneity of the findings. The results were presented as forest plots. The analysis was implemented in R version 4.01.

## Results and discussion

### Literature review of what is known based on eligible studies

To begin with, Shanes et al. (17) analyzed 16 placentas from pregnant women infected with SARS-CoV-2. Fourteen were delivered at term (37–40 weeks), one was born at 34 weeks, and the last was a 16-week intrauterine fetal demise. Several features of MVM and FVM were common in all 12 out of 15 cases, such as fetal chorangiosis, villous infarction, and decidual arteriopathy. Interestingly, none of the placentae in their study was positive for SARS-CoV-2, and there was no account of maternal or neonatal death, and likewise, all infants’ nasopharyngeal and throat swabs were negative for SARS-CoV-2 (17). One possibility is that at term, placenta expression for angiotensin-converting enzyme 2 receptor (ACE2) and transmembrane serine protease 2 (TMPRSS2) is low, and the placental inflammation might be systemic rather than local.

It is worth noting that the expression of ACE2 protein and TMPRSS2 are prerequisites for the SARS-CoV-2 entry. Taglauer et al. (18) reported that both ACE2 and SARS-CoV-2 spike (S) glycoprotein co-localize the outer syncytiotrophoblast (STB) layer of placenta villi that juxtaposed at the maternal-fetal interface, however, when compared to the non-infected group, ACE2 expression was downregulated. This could be on account of the placentas included in Taglauer et al. (18) study consist of a placenta cohort from the third trimester COVID-19 positive pregnancies. The finding of decreasing ACE2 receptors at the third trimester is consistent with other eligible studies found in our search (19, 20). This might suggest that SARS-CoV-2 – placenta infection might have an alternative route, or placenta at term provides a remarkable protective role in developing fetus against SARS-CoV-2 infection. However, further validation is required with more literature/report cases before a solid conclusion can be drawn.

Likewise, Pique-Rogue et al. (21) investigated whether ACE2 and TMPRSS2 were expressed in human placenta throughout the pregnancy windows (i.e., first, second, and third trimesters), including the chorioamniotic membranes in the third trimester by using a publicly available single-cell RNA-sequencing (scRNA-seq) data. They found that both ACE2 and TMPRSS2 were minimally expressed on the placenta in the first trimester and chorioamniotic membrane (21). One limitation of using scRNA-seq is the difficulty of generating high-quality single-cell suspension containing multinucleated cells such as STB, affecting the scRNA-seq analysis output. STB comprises cells that participate in fetal-placenta circulation (21, 22). To address this, the authors prepared single-nuclear suspension from frozen placenta tissue and performed a single-nuclear RNAseq. As expected, snRNA-seq revealed that co-expression of placenta ACE2 and TMPRSS2 was improbable. Similarly, mining two microarray datasets obtained from previous studies revealed that the co-expression of the two proteins was negligible in the third trimester (21).

Beesley et al. (20) analyzed the expression of ACE2 and TMPRSS2 on fetal samples at both transcriptional and translation levels; only two sample types (kidney and intestine) manifested co-expression of both proteins from the second-to-third trimester. These findings were comparable to the conclusion of using publicly available scRNA-seq data. It is plausible that fetal SARS-CoV-2 infection might occur via the gastrointestinal tract since the fetal intestine is directly exposed to amniotic fluid via fetal swallowing (20). This can be possible only if there is a presence of SARS-CoV-2 in the amniotic fluid. However, few studies did not detected SARS-CoV-2 in the amniotic fluid (23, 24). Therefore, this path might be rare as well. Furthermore, there was no evidence of ACE2 and TMPRSS2 proteins co-expression in the placenta in the second trimester to term (20). The lack of evidence for ACE2 and TMPRSS2 co-expression in single-cell and single-nuclei transcriptomic analysis in these studies indicates a low likelihood of placenta infection and vertical transmission at term.

In their study, Bloise et al. (25) investigated the expression pattern of ACE2 and TMPRSS2 in the placenta across pregnancy trimesters. The key finding was that the expression of ACE2 and TMPRSS2 decreases as gestation progresses, suggesting differential susceptibility to placenta SARS-CoV-2 infection during the trimester of pregnancy. Similarly, Colson et al. (26) isolated trophoblast from non-pathologic human placenta, and infected the culture with SARS-CoV-2, they found that after 4 days post-infection, viral particles were not detected in the supernatant or within the trophoblast. A similar result was observed in TMPRSS2 transfected STB infected with SARS-CoV-2, highlighting that TMPRSS2 alone was insufficient to induce SARS-CoV-2 infection (26). This indicates that the cell endocytosis mechanism for SARS-CoV-2 to gain entry does not occur in the infected cultured trophoblast or transfected cells and that, at term, the susceptibility of the placenta to SARS-CoV-2 might be extremely low (26).

ACE2 are also reported to be expressed in extra-placental tissues. Faure-Bardon et al. (27) reported that ACE2 were expressed in the maternal-fetal interface, including cytotrophoblast (CTB) and STB of placenta, fetal testis, gastrointestinal tract and kidney samples. These findings suggest that SARS-CoV-2 is able to cross the placenta via either vertical transmission or blood-borne transmission and possibly infect fetal organs at any gestational age. Furthermore, Ouyang et al. (28) also reported that in addition to TMPRSS2 and ACE2, placenta trophoblast expressed Furin receptor as well, which could mediate SARS-CoV-2 entry. Dong et al. (8) also reported that in addition to ACE2, CD147 and Glucose Regulating Protein 78 (GRP78) were highly expressed in the placenta maternal-fetal interface of pregnant women diagnosed with SARS-CoV-2 infection. Both CD147 and GRP78 can also mediate SARS-CoV-2 entry into cells (8). Understanding the mechanism of these non-canonical receptors acting as an alternating route for SARS-CoV-2 is of interest. The role of CD147 and GRP78 in placenta function and in SARS-CoV-2 had been discussed elsewhere (29–31).

Fenizia et al. (32) used molecular biology techniques such as real-time PCR to analyze the presence of SARS-CoV-2 genome in different maternal, fetal and maternal-fetal interface collected samples. Additionally, they investigated the placental inflammatory response to SARS-CoV-2. The authors detected SARS-CoV-2 genome in maternal plasma, vaginal swabs, placental tissue, breast-milk sample, cord plasma in the samples of two out of 31 pregnant women. Furthermore, there was an upregulation in inflammatory gene activation, especially in adaptive immune activation, innate immune cells, Toll-like receptors (TLR), cytokine and chemokine expression in three patients’ placental samples (32). The upregulation of genes involved in immune activation might be responsible for cytokine storms observed in COVID-19 positive patients, as seen in Garcia-Flores findings (33). The presence of SARS-CoV-2 genome in some maternal-fetal interface tissue (i.e., one umbilical cord and two at-term placenta samples from 31 mothers), suggested that vertical transmission was likely to occur but low possibility.

Using IHC and *in situ* hybridization techniques, Menter et al. (23) showed that microvasculopathy was common in placentas of SARS-CoV-2 positive women. Placentas at-term from SARS-CoV-2-positive pregnant women diagnosed with mild symptoms or no symptoms of COVID-19 prior to child delivery presented prominent signs of FMV, MVM, lymphohistiocytic villitis and intervillositis. These findings support the hypothesis that SARS-CoV-2 can cause an inflammatory response in infected placentas, which can lead to the demise of the developing fetus (23).

Similar to Pique-Roger et al. (22) bioinformatics analysis study on SARS-CoV-2 entry receptor expression on human placenta, Cui et al. (34) reported that ACE2 and TMPRSS2 were spatially distributed in human trophectoderm, first- and second-trimester placentas using bioinformatics tools on publicly scRNA-seq, as opposed to Pique-Roger et al.’s (22) study whose primary focus was on receptors co-localization. ACE2 and TMPRSS2 expression was observed in CTB, STB, and extravillous trophoblast (EVT) cells at different placenta ages from both the scRNA-seq data and IHC (33). Placenta and decidua are the main maternal-fetal interface, Li et al. (35) collected online scRNA-seq data to evaluate cells that express ACE2 in the maternal-fetal interface and fetal organs. The authors revealed that ACE2 were highly expressed in the maternal-fetal interface including the stromal cells and perivascular cells of decidua, villous CTB and STB in the placenta. Furthermore, Li et al. (35) also observed that both ACE2 and TMPRSS2 were spatially expressed in human fetal organs, including cardiomyocytes (CM), macrophages and smooth muscle cells, pericytes, hepatocytes, airway epithelial cells. The expression of ACE2 and TMPRSS2 pose a high risk of neonatal infection.

Furthermore, Lu et al. (36) also reported the expression of ACE2 on fetal organs such as the adrenal gland, heart, and kidney but not in airways epithelial cells and fetal-placenta interface, thus suggesting the possibility of low vertical transmission during pregnancy. The absence of ACE2 on fetal airway epithelial cells opposed the findings of Li et al. (35). These discrepancies might be attributed to the difference in physiological change of cell types along the trimesters of pregnancy used for the transcriptomic studies and the possibility of differences in snRNA-seq datasets used in each downstream study analysis.

Smithgall et al. (37) reported evidence of maternal and fetal vascular malperfusion in the placenta from SARS-CoV-2 infected mothers in their third trimesters. These were subchorionic thrombi, intervillous thrombi, fetal thrombotic vasculopathy and chorangiosis. Facchetti et al. (38) screened women who delivered during the early phase of the COVID-19 pandemic for SARS-CoV-2 N and S proteins and their babies. They found the presence of SARS-CoV-2 antigen, RNA and particles in the placenta, maternal inflammatory cells and multiple fetal villous cellular subsets in COVID-19 positive mothers, thus supporting the hypothesis of SARS-CoV-2 transmission from mother to fetus either during pregnancy or at birth. Consistent with Facchetti et al.’s (38) findings, using IHC and *in situ* hybridization staining methods, Rocha et al. (39) reported the presence of SARS-CoV-2 proteins in formalin-fixed placenta tissue, lung biopsies but not in kidney biopsies in COVID-19 positive patients. Schoemarker et al. (40) diagnosed placental inflammation caused by SARS-CoV-2 based on viral detection on STB, and maternal and neonatal samples using the qPCR method. The key finding was that there were similar histopathological placenta findings as previously reported in the literature (23, 41, 42). In addition, there was prominent CD20+ B-lymphocytes infiltration which had not been described in histiocytic intervillositis (39). The authors also reported that maternal breast milk and all the neonatal samples were negative for SARS-CoV-2, but the neonates showed a need for care in the neonatal intensive care unit (NICU).

Debelenko et al. (43) reported STB damage leading to placenta trophoblast necrosis accompanied by mixed intervillositis and perivillous fibrin deposition in placenta of COVID-19 positive women. Similar to Debenko et al.’s findings of trophoblast necrosis, Garrido-Pontnou et al. (44) also reported that only about 4.5% of placentas collected from women with COVID-19 showed similar features of trophoblast necrosis and intervillous space collapse. Although the rate of SARS-CoV-2 infection in the placenta might be low, it suggested that trophoblast necrosis, along with inflammatory infiltration as seen in emerging studies, was one of the hallmarks of SARS-CoV-2 infection of the placenta, and this was associated with fetal demise (43).

Additionally, Wu et al. (45) reported that there was increased CD14+ macrophage infiltration, interferon-γ induced protein 10 (IP-10) and monocyte induced by gamma interferon (MIG) cytokines expression in term placenta from pregnant women recovering from COVID-19. The increased production of these types of chemokines within the local inflammatory lesions can induce a Th1-mediated antiviral immune response, leading to macrophage recruitment and activation for virus clearance (45). Another key finding was the reduction of plasma IL-12 secretion in pregnant patients who had recovered, indicating the activation of anti-inflammatory response might have already occurred during infection (45). Likewise, Zhao et al. (19) reported that pregnant women who had recovered from COVID-19 had decreased levels of IL-1ra and monocyte chemoattractant protein 1 (MCP-1), which was expected. However, the count was low in memory B cells, type 2 T cells (Th2) and follicular T helper cells (Tfh17) (19). Placentas from this category of pregnant women showed no co-expression or co-localization of ACE2 and TMPRSS2. This might simply mean that direct SARS-CoV-2 infection in the placenta is unlikely to occur since the entry receptors diminish. Thus local placenta inflammation will be negligible via the canonical SARS-CoV-2 entry receptors, and as such possible direct effect on fetal demise might be negligible or rare (19).

Mulvey et al. (46) investigated the placenta pathology of five full-term births born to COVID-19 positive pregnant women. All five showed evidence of FVM, such as thrombosis, and the result from immunostaining for viral RNA and viral spike protein on the placenta was negative. This result suggests that the observed thrombosis was due to a systemic rather than a local effect of virus infection (45).

In a retrospective cohort study, Patberg et al. (47) reported that placenta collected from COVID-19 positive women delivering at term showed histopathological features of FVM and villitis of unknown etiology. Whether the placenta can re-produce viral particles in cell suspension is a hot topic, which would buttress our understanding of the virus replicative capacity. Interestingly, Tallarek et al. (48) revealed that there was an inefficient replication capacity of SARS-CoV-2 in placenta explant cultures. Moreover, the authors assessed whether there was SARS-CoV-2 specific T cell-mediated immunity in pregnant women and the cord blood to attempt the puzzle on fetoplacental transmission. The results showed that specific cell-mediated immunity found in maternal blood was absent in neonatal cord blood, which revealed that the virus did not cross vertically to the fetal side (47). In addition to Hofbauer cells and T cells specific response during placental inflammation due to SARS-CoV-2 infection, Husen et al. (49) also reported specific distinct CD20+ B cells infiltration along with features of placental abnormalities such as MVM, FVM, chorioamnionitis and/or multifocal low-grade villitis with unknown etiology. These placental signatures might affect fetal well-being.

Moving forward, Bouachba et al. (50) reported that SARS-CoV-2 infection in the placenta induced massive inflammatory lesions and necrosis in the placenta. This was associated with poor fetal outcomes, including death, intrauterine growth restriction, and even premature neonates outcomes. There were also intervillous thrombi, massive perivillous fibrin deposit, CHI accompanied by macrophage infiltration, consistent with other studies (50, 51). However, Rebutini et al. (52) did not observe a significant difference in Hofbauer cells infiltration with changes in placenta morphology from both the COVID-19 positive and control groups, but there was observed features of CHI, MVM and FVM. Fetal vascular thrombosis happens to be one of the most common FVM observed, and this is likely to be associated with the worst neonatal outcomes, such as preterm birth and even death (51). Similarly, Gulersen et al. (53) also observed no statistical difference in the histopathological features of placentas from women with or without symptoms of SARS-CoV-2 infection and women with historical pregnancy complications such as gestational diabetes, preeclampsia, and intrapartum fever. However, there were a few limitations in Gulersen et al.’s study, the placenta was not tested for SARS-CoV-2, so it was difficult to tell whether the findings were a result of direct SARS-CoV-2 placenta infection or an indirect effect from maternal immune-physiological response to the virus (52).

Santana et al. (3) reported that vertical transmission was highly unlikely to occur and there was no significant difference in morphological placenta findings (i.e., fetal vascular malperfusion) among their study groups. Further, Sinaci et al. (24) analyzed 48 valid samples comprising the placenta, cord blood, amniotic fluid and vaginal swabs from COVID-19 pregnant women in their third trimester, and found that only one placenta and vaginal swab samples were positive for SARS-CoV-2.

In a cohort study of 66 SARS-CoV-2 positive pregnant women in their third trimester, Mourad et al. (54) categorized the women into severe and mild or asymptomatic groups and found that antiviral Interferon-induced transmembrane (IFITM1 and IFITM2), ACE2 and not TMPRSS2 or Furin were highly expressed on the placenta of severe COVID-19 pregnant women. IFITMs are antiviral innate immune response genes. However, there was no correlation between the expression of IFITIMs and SARS-CoV-2 entry receptor to placental histopathology (54). In neonates, fetal plasma was negative to anti-SARS-CoV-2- IgG, IgA or IgM antibodies, supporting that vertical transmission was rare and the placenta might exert a sort of resistance to SARS-CoV-2 infection (54).

On the contrary, Halici-Ozturk et al. (55) assessed vertical transmission in PCR-positive women and found that all valid placenta and curettage materials tested negative for SARS-CoV-2, suggesting that there was no evidence for SARS-CoV-2 transmission in early pregnancy based on their study. Similarly, Edlow et al. (56) also reported no detection of SARS-CoV-2 viral load in the blood cord from neonates born to SARS-CoV-2 positive pregnant women and thus no evidence of vertical transmission.

Tight junction (Tjs) proteins, placental vascular endothelial (VE)-cadherins and claudin-5 provide the molecular framework that facilitates the cell-to-cell adhesion complex that is crucial for placenta barrier function. In consequence, disruption of this unique framework might increase vascular leakage and permeability. Flores-Pliego et al. (57) reported that VE-cadherins and claudin-5 expression decreases in placenta decidua and villi in women with severe COVID-19, accompanied by Hofbauer hyperplasia, thrombosis with the remodeling of the villi and infarcts with intervillositis. Thus, it is plausible that severe COVID-19 during pregnancy might induce placental endothelial leakage due to loss of adherence function, promote inflammation and induce shock.

Further, Fahmi et al. (58) reported that SARS-CoV-2 propagated efficiently in placenta precision-cut slices (PCSs) samples from six out of seven donors. This correlated with the extent to which ACE2 was expressed on the placenta samples (57). The authors also further reported the presence of SARS-CoV-2 viral RNA and/or viral proteins on hofbauer macrophages and in different regions of the placenta explants. Surprisingly, this event did not trigger pro-inflammatory cytokines production but rather induced interferon-gamma expression by the trophoblast, which is consistent with Mourad et al. findings (54). As much as placenta PSCs help understand early phases of placenta – SARS-CoV-2 infection biology, it did not consider the placenta barrier and the outcome influenced by immune components (57).

Recently, Garcia-Flores et al. (33) reported that pregnant women infected with SARS-CoV-2 had unique inflammatory response pattern at the maternal-fetal interface, governed by the maternal T cells population and fetal stroma cells. There were increased maternal cytokines (such as IL-8, IL-10, and IL-15) in the systemic circulation. Likewise, neonates born to women infected with SARS-CoV-2 also showed an increase of IL-8 compared to those born to control mothers. Apart from the cytokine response observed, women infected with SARS-CoV-2 showed a significant reduction in CD4+ and CD8+ T cells. Although the severity of the disease did not solely drive such changes, none of these changes were observed in neonates born to COVID-19 positive mothers (58).

Glynn et al. (59) showed that these placenta lesions on the maternal and fetal side were time-dependent across the pregnancy window in delivery and disease severity. The immune response to SARS-CoV-2 is an interesting topic. Hsieh et al. (60) reported that immune hemostasis was not compromised by SARS-CoV-2 infection, as most of the predominant innate immune cells population in all subjects studied were tolerogenic. Most of these cells were primed CD14+ dendritic cells and tolerogenic myeloid dendritic cells. This suggests that SARS-CoV-2 does not jeopardize immunity at the maternal-fetal interface.

#### SARS-CoV-2 entry receptors expression and maternal-fetal transmission

Our selection strategy was summarized in Figure 1. Table 1 summarized ACE2/TMPRSS2 differential expression in the placenta at different pregnant windows and the possibility of vertical transmission. This study identified forty-five studies that met the inclusion criteria reporting 1280 human placentas analyzed by qPCR, IHC, Immunofluorescence (IF) and/or *in situ* hybridization to investigate the expression of SARS-CoV-2 entry receptor, placenta pathology and evidence of vertical transmission. Additionally, two out of the 45 identified studies utilized bioinformatics tools to screen 11,112 placenta-derived cells from a publicly available database to investigate the expression of SARS-CoV-2 entry receptors at different trimesters. ACE2 and TMPRSS2 proteins were abundantly expressed in the placenta during the first trimester, and this expression diminished along with the pregnancy window (19, 21, 23, 25, 26) (see Table 1).

**TABLE 1:** Studies highlighting the status of vertical transmission in SARS-CoV-2 infected placenta samples at different pregnancy windows (*n* = 45); 1280 primary human placenta; 11112 derived placenta cells.

S/N	Authors	Year	Country of study	Sample size (Human) *	SARS-CoV-2 entry receptor expression/or co-localization on placenta	Method of detection	Stage of pregnancy	Authors finding on vertical transmission	Maternal – fetal transmission
1	Shanes et al. (17)	2020	United States	16 (all were from maternal positive SARS-CoV-2)	None of the placenta in this study was tested for SARS-CoV-2 entry proteins	IHC, qPCR	Third trimester	Placenta infection of SARS-CoV-2 are related to maternal infection rather than fetal infection	No
2	Taglauer et al. (18)	2020	United States	25 (15 from maternal positive SARS-CoV-2)	Presence of SARS-CoV-2-spike glycoprotein and ACE2 co-localize at the outer STB layer of placenta villi	IHC	Third trimester	No evidence of fetal transmission	No
3	Pique-Regi et al. (21)	2020		32	Co-transcription of ACE2 and TMPRSS2 is negligible, though there is increased expression of alternate SARS-CoV-2 receptor like BSG	Single cell/nucleic RNA sequencing	Throughout pregnancy and at term	Placenta minimally expressed SARS-CoV-2 entry receptors. Not likely of vertical transmission. more studies is required to understand placental resistance to SARS-CoV-2 infection	No
4	Fenizia et al. (32)	2020	Italy	31 (2 SARS-CoV-2 positive placentas)	NA	qPCR	Term (third trimester)	Possibility of vertical transmission	Yes
5	Menter et al. (23)	2021	Switzerland	5 (all positive)	ACE2 are weakly expressed	qPCR	Third trimester	Findings support the hypothesis of SARSCoV-2 invading the placenta, but there is no evidence of transplacental transmission. As umblical cord and amniotic fluid were negative to SARS-CoV-2	No
6	Cui et al. (34)	2021		9 (positive not given) and (1260 derived placenta cells for bioinformatic studies)	ACE2 and TMPRSS2 were expressed on placenta	Bioinformatics and IHC	Throughout pregnancy	Possibility of intrauterine fetal infection (transplacenta transmission), but rare and low.	Yes
7	Li et al. (35)	2020		32	ACE2 and TMPRSS2 are expressed in stroma cells, perivascular cells of decidua, CTB and STB of the placenta	Bioinformatics analysis such as scRNA-seq	First trimester	Possibility of vertical transmission. The authors also suggested that the expression of ACE2 and TMPRSS2 might increase along the trimesters	Yes
8	Lu et al. (36)	2020		9852 derived placenta cells	Few trophoblast expresses ACE2 receptors (*n* = 9/9852)	Bioinformatics analysis such as scRNA-seq		Low possibility of vertical transmission	Yes
9	Smithgall et al. (37)	2020	United States	76 (51 from maternal positive SARS-CoV-2)	NA	IHC, ISH, PCR	Third-trimester placentas	No comment	NC
10	Facchetti et al. (38)	2020	Italy	15 (all were from maternal positive SARS-CoV-2)	No report on ACE2 expression, but there was presence of SARS-CoV-2 S and N protein in placenta sample (*n* = 1/15)	IHC, ISH		Support the hypothesis of possible transverse transmission	Yes
11	Schoenmarkers et al. (40)	2021	–	1 (all was SARS-CoV-2 positive placenta)	Presence of SARS-CoV-2 conserved protein in STB	qPCR, IHC, ISH	Third trimester	No evidence of vertical transmission	No
12	Bloise et al. (25)	2021	Canada	87 (SARS-CoV-2 positive placentas were not given)	High ACE2 and TMPRSS2 expression in first trimester placenta than in second trimester, preterm and term placenta	qPCR, and Next generation sequencing or RNA sequencing	Throughout trimesters	No increase in mRNA expression of ACE2 and TMPRSS2 at the maternal-fetal interface, suggesting an unlikely event of vertical transmission across the gestation period.	No
13	Faure-Bardon et al. (27)	2021	–	7 (1 SARS-CoV-2 positive placentas)	ACE2 and TMPRSS2 present on placenta	NA	Second – third trimester	Supports the possibility of vertical transmission	Yes
14	Debelenko et al. (43)	2020	United States	75 (All were from maternal positive SARS-CoV-2)	NA	PCR, IHC, ISH	NA	All neonates born to COVID-19 pregnant women were negative for SARS-CoV-2	No
15	Wu et al. (45)	2021	China	11 (6 SARS-CoV-2 positive placentas)	ACE2 and TMPRSS2 present on placenta	qPCR and IHC	NA	No evidence of transverse transmission, but transverse natural immunity is possible	No
16	Valdespino-Vazquez et al. (61)	2021	Mexico	NA	SARS-CoV-2 conserved gene present on placenta	qPCR, IHC, IF	First trimester	There is possibility of congenital SARS-CoV-2 infection during the first trimester of pregnancy	Yes
17	Cribiu et al. (62)	2021	–	37 (50% were SARS-CoV-2 positive placentas)	NA	qPCR, IHC	Most are Term	No vertical transmission observed. Though umbilical cord blood was not tested for SARS-CoV-2	No
18	Patberg et al. (47)	2021	United States	77 (All were from maternal positive SARS-CoV-2)	NA	PCR	Term	All neonates born to COVID-19 positive mothers tested negative after birth	No
19	Dong et al. (8)	2021	Wuhan, China	9 (*n* = 7/9 are positive)	ACE2 were expressed on the maternal side of the placenta, but low at the villous matrix and interstitial blood vessel have low expression of ACE2. CD147 (also known as Basigin) is a novel route for SARS-CoV-2, expressed in the surface of STB of the placenta	qPCR, IHC	NA	Very low probability of vertical transmission	Yes
20	Tallarek et al. (48)	2021	Finland	42 (21% are positive)	NA	qPCR, IHC	All trimesters	Vertical transmission is rare, likely due to inefficient viral replication in placental tissues	No
21	Blasco Santana et al. (3)	2021	Spain	29 (1 from maternal positive SARS-CoV-2)	NA	IHC, qPCR	Third trimester	Vertical transmission is rare	Yes
22	Schwartz et al. (51)	2021	Multi-countries	22 (All were from maternal positive SARS-CoV-2)	Positive detection of SARS-CoV-2 in Hofbauer cells (*n* = 4/22), STB (*n* = 21/22), villous capillary (*n* = 2/21)‘	IHC, qPCR	NA	Possible vertical transmission. SAS-CoV-2 can infect beyond the trophoblast into the villous stroma, but the sample is small and this might not be associated with fetoplacental transmission. The author further states that transplacental infection might occur in the absence of Hofbauer cells and endothelial involvement	Yes
23	Bouachba et al. (50)	2021	Brazil	82 (Positive *n* = 5/82 with severe outcome)	ACE2 expressed on trophoblast	IHC	Not specified	No sign of maternal-to-fetus infection in postmortem examination (*n* = 3/5). Preterm birth was seen in *n* = 2/5, and this cannot be explained by virus transmission but rather by placental lesion	No
24	Sinaci et al. (24)	2021	Turkey	Exact number not given (1 SARS-CoV-2 positive placentas)	NA	PCR	NA	Vertical transmission might be possible but in this case, it is rare and via the vaginal during delivery	Yes
25	Rebutini et al. (52)	2021		9 (1 from maternal positive SARS-CoV-2)	ACE2 and TMPRSS2 were express on placenta	IHC	Second and third trimester	No comments	NC
26	Garrido-Pontnou et al. (44)	2021	Spain	9 (All were from maternal positive SARS-CoV-2)	NA	IHC, ISH, PCR	NA	Two neonates born to COVID-19 positive mothers samples were positive for SARS-CoV-2, suggesting the possibility of vertical transmission	Yes
27	Mourad et al. (54)	2021	United States	43 (All were from maternal positive SARS-CoV-2)	placental ACE expression increased in severe COVID-19 patients	IF	Third trimester	COVID-19 severity during pregnancy does not correlate with fetoplacental transmission	No
28	Halici-Ozturk et al. (55)	2021	Turkey	Not specified	NA	PCR	First trimester	No evidence of vertical transmission	No
29	Gulersen et al. (53)	2020	United States	50 (All were from maternal positive SARS-CoV-2)	NA	PCR	Third trimester	Unlikely to occur, all neonates samples test negative	No
30	Flores-Pliego et al. (57)	2021	Mexico	11 (All were from maternal positive SARS-CoV-2)	NA	PCR, IF	Second-third trimester	No comment	NC
31	Husen et al. (49)	2021	Netherlands	39 (were from 17 maternal positive SARS-CoV-2)	NA	PCR	NA	placental SARS-CoV-2 infection can result to placental damage, triggering inflammation and compromising the maternal-fetal interface. Thus impaired placenta function increased risk for fetal demise and this is not associated to vertical transmission	No
32	Colson et al. (26)	2021	Belgium	31 (all were from from maternal positive SARS-CoV-2)	Placenta at term do not express ACE2 and TMPRSS2	PCR, IHC, ISH	Term	Placental are not likely to be infected by SARS-CoV-2 at term. Vertical transmission might not occur because of the tight immunomodulatory mechanism of the placenta	No
33	Zhao et al. (19)	2021	China	20 (All from maternal recovering from SARS-CoV-2)	ACE2 expression was high during the first trimester, and gradually decreases in second and third trimesters			ACE2 and TMPRSS2 are not usually co-expressed in the placenta, its less likely SARS-CoV-2 can cause infection on the fetus via these routes. Thus, vertical transmission would be very rare via the ACE2/TMPRSS2 route	No
34	Morotti et al. (65)	2021	Italy	1 (All from from maternal positive SARS-CoV-2)	NA	PCR, IHC	Term	No evidence of SARS—CoV-2 vertical transmission	No
35	Beesley et al. (20)	2021		NA	ACE2, TMPRSS2 expression is very low at term	PCR, IHC	NA	An interesting finding suggesting possible transverse transmission via the GIT, because the GIT co-express both SARS-CoV-2 entry receptors proteins. This susceptibility may be present from late second trimester. Their studies also found out that placenta at term does not co-express these two receptors, and as such transmission might not occur via these route.	No
36	Alouini et al. (66)	2022	France	30 (1 was positive for SARS-CoV-2)	NA	PCR	Term	Maternal IgG against SARS-CoV-2 were present in 20/41 (48.8%) cord blood samples, In maternal blood; IgG was positive 20/37 cases (54%), and IgM positive for 4/23 cases (17%). The presence of neonatal IgG against SARS-CoV-2 reflect why fetus is rarely infected by the virus. Vertical transmission of the virus is rare	Yes
37	Celik et al. (67)	2022	Turkey	20 (None was positive)	NA	PCR, IHC	Term	Neither vertical transmission nor placenta infection were detected in all women included in this study. SARS-CoV-2 was not detected at term placenta. SARS-CoV-2 was not detected in neonates nasopharyngeal swab collected immediately after delivery. vertical transmission of SARS-CoV-2 did not occur	No
38	Dubucs et al. (68)	2022	France	50 (All were from maternal positive SARS-CoV-2)	NA	PCR	Term	Rare, and might be unlikely to occur at third trimester. Placenta lesion does not correlates with maternal SARS-CoV-2 infection at term pregnancy	No
39	Fahmi et al. (58)	2021	Switzerland	Not given	ACE2 expression was in high amount	PCR	Term	No comment	NC
40	Garcia-Flores et al. (33)	2022	United States	Not given	NA	IHC, PCR	Term	Unlikely to occur	No
41	Glynn et al. (59)	2022	United States	90 (All were from maternal positive SARS-CoV-2)	NA	PCR	Second trimester	No comment	NC
42	Shook et al. (63)	2021	United States	68 (38 SARS-CoV-2 positive, 30 SARS-CoV-2 negative women)	ACE2 and TMPRSS2 were expressed. ACE2 was not impacted by fetal sex or maternal exposure to SARS-CoV-2. But TMPRSS2 does	PCR	Not given	Maternal exposure to SARS-CoV-2 have a significant impact on the expression of ACE2 and TMPRSS2 in only male placenta. Male fetal influences placental susceptibility to SARS-CoV-2 infection, and possibly aggravate the risk for fetoplacental transmission, which of course dependent on the age of the placenta	Yes
43	Vivanti et al. (69)	2020	France	1 (positive)	NA	PCR, IHC	Term	Vertical transmission is possible	Yes
44	Hsieh et al. (60)	2022	United States	NA	NA	Immunophenotyping, flow cytometry	Term	No comment: cord blood or blood tissue from infant were not investigated	NC
45	Edlow et al. (56)	2020	United States	88 (44 positive for SARS-CoV-2, the remaining negative)	ACE2 and TMPRSS2 were expressed on the examined placenta sections	PCR		No evidence of vertical transmission	No

*Placentas from SARS-CoV-2 positive pregnant women does not necessary translates to positive fetotransmission. NC, no comment.

**TABLE 2:** Pathology features of SARS-CoV-2 infected placenta from 12 selected studies.

Features of placenta pathology	*n* (n/N)	%
Chronic histiocytic intervillositis and trophoblast necrosis*	26 (0.0600)	6.0
Massive perivillous fibrin deposit and intervillosis thrombi formation	4 (0.0092)	0.9
**(a) Features of Maternal Vascular Malperfusion (MVM)**		
Intervillous thrombosis*	10 (0.0231)	2.3
Increase of intervillous fibrin	4 (0.0092)	0.9
Infarction*	14 (0.0323)	3.2
Decidual vasculopathy or arteriopathy*	14 (0.0323)	3.2
Subchorionic thrombus	9 (0.0207)	2.1
Villous agglutination	21 (0.0484)	4.8
Distal villous hypoplasia/Accelerated villous maturation	3 (0.0069)	0.7
Absence of spiral artery remodeling	6 (0.0138)	1.4
At least one form of MVM *	17 (0.0392)	3.9
**(b) Features of Fetal Vascular Malperfusion (FVM)**		
Chorangiosis *	12 (0.0277)	2.8
Thrombi in the fetal circulation or thrombic vasculopathy*	22 (0.0508)	5.1
Villous stromal vascular karyorrhexis*	2 (0.0046)	0.5
Segmental avascular villi*	20 (0.0461)	4.6
Intramural fibrin deposition *	13 (0.0300)	3.0
Fetal vascular thrombosis in the umbilical cord	5 (0.1154)	1.2
Multifocal low grade villitis of unknown etiology	17 (0.0092)	3.9
High grade villitis	6 (0.0138)	1.4
Chorioamnionitis	14 (0.0323)	3.2
At least one form of FVM*	16 (0.0369)	3.7

Total number of placenta (N = 433) were identified. *More than one studies reporting similar findings.

Maternal-fetal transmission of SARS-CoV-2 during the three trimesters and postpartum remains debatable and a huge public health concern. Our study identified 24/45 (53.34%) studies that showed no evidence of vertical transmission, 15/45 (33.33%) support the hypothesis of vertical transmission but was very rare, and most likely to occur during the first trimester via the ACE2/TMPRSS2 route (Table 1). Six studies (13.33%) were indecisive and had no comment on whether vertical transmission occurred or not. Vertical transmission did not occur because the ACE2/TMPRSS2 were not co-expressed in the placenta at term, but transverse natural immunity might be possible. One study suggested the possibility of vertical transmission via the gastrointestinal tract (GIT) in the late second trimester. This was attributed to the co-expression of ACE2 and TMPRSS2 within the GIT and not in the placenta (20).

**FIGURE 2:**
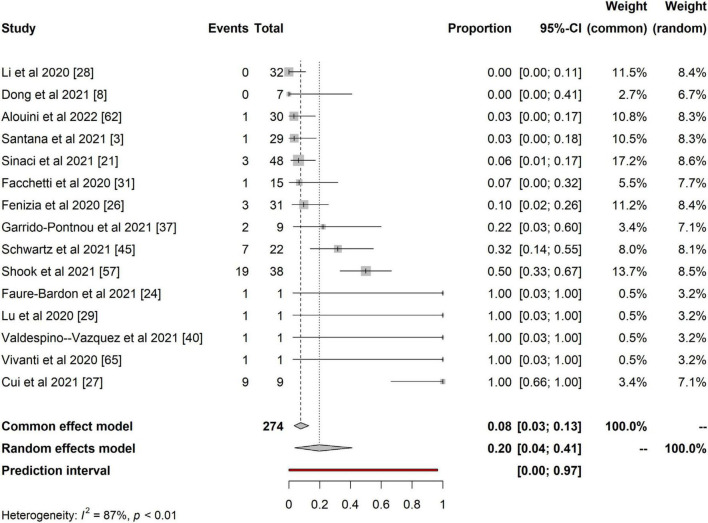
Pooled proportional estimates for investigating evidence of fetoplacental transmission of SARS-CoV-2.

Furthermore, we present the quantitative analysis of the 15 studies that reported evidence of fetoplacental transmission of SARS-CoV-2. Among the studies analyzed, the prevalence of fetoplacental transmission of SARS-CoV-2 was 0.20 (95% CI: 0.04 – 0.41, *I^2^* = 87%), as shown in Figure 2. The prediction interval of fetoplacental transmission of SARS-CoV-2 was from 0 to 0.97, with 95% confidence. Although we estimated a pooled of 1 in 5 fetoplacental transmissions of SARS-CoV-2 from COVID-19 infected mothers based on 15 studies, the prediction interval suggested a null to a very low effect. This is not surprising given the presence of high inconsistency between studies (*I^2^* = 87%). For example, in their neonatal samples of 48 newborns, Sinaci et al. (24) reported that only one tested positive for SARS-CoV-2 RNA and two were positive for IgG-M antibodies. These results suggest that vertical transmission is extremely low and rare, and exposure to SARS-CoV-2 occurs in the uterus during perinatal life. Schwartz et al. (51) further reported that the placenta macrophage, Hofbauer cells, were involved in SARS-CoV-2 infection in the placenta with the presence of chronic histiocytic intervillositis (CHI). Four out of the 22 placentae stained positive for Hofbauer infected SARS-CoV-2, confirming that indeed the virus can cross the placenta but low and induce local inflammation that leads to Hofbauer hyperplasia (51). In a case study, Valdespino-Vazquez et al. (61) reported that congenital SARS-CoV-2 infection is possible in the first trimester and that the fetal lung and kidneys are potent targets. This intrauterine transmission occurs in the first trimester and is associated with fetal demise and miscarriages. Cribiu et al. (62) reported the presence of placenta SARS-CoV-2 infection in almost half of the pregnant women infected with SARS-CoV-2 in their study. But, there was no association of SARS-CoV-2 with any distinctive pathological features, and maternal and fetal outcomes in the third trimester, suggesting that intrauterine transmission of SARS-CoV-2 in neonates might be an independent phenomenon and unlikely to occur at late pregnancy age. However, the authors did not investigate the umbilical cord for anti-SARS-CoV-2 antibodies testing.

Whether fetal sex influences placenta susceptibility to SARS-CoV-2 is an emerging focus. Recently, Shook et al. (63) (which happens to be the first report on this subject) showed that maternal SARS-CoV-2 infection was associated with increased correction between ACE2 and TMPRSS2 expression in male placental only. This suggests that sex differences might influence placenta vulnerability to SARS-CoV-2 and possibly, in fetoplacental transmission; however, no study has shown this possibility.

Taken together, this might suggest that SARS-CoV-2 could have severe impacts on the placenta in early pregnancy, and its impact in the third trimester is systemic rather than local placenta infection. The question about vertical transmission is still debatable. It is expected that large population clinical studies will address the concern about vertical transmission in neonates in the future.

#### Placenta pathology

We identified 12 studies reporting the impact of SARS-CoV-2 on placenta pathology that met the inclusion criteria (section “Study selection and criteria”). A total of 433 placentas were identified from these twelve studies, out of which 26 (6.0%) placentas showed signs of CHI and trophoblast necrosis. We also found features of maternal vascular malperfusion (MVM), 57/433 (13.1%) and fetal vascular malperfusion (FVM), 81/433 (18.7%), which included: Decidual vasculopathy (*n* = 14; 3.2%), intervillous thrombosis (*n* = 10; 2.3%) and infarction (*n* = 14; 3.2%) were common features of MVM and thrombotic vasculopathy (*n* = 22; 5.1%) and chorangiosis (*n* = 12; 2.8%) were common features of FVM observed in our study (see Table 2).

The placenta is an immunologically privileged organ that provides nutrients and protection to developing fetuses. Lately, several studies have shown that the placenta is a target for viral infections such as the Zika virus, which ultimately induces pathologies similar to the present study’s findings (9, 23). Interestingly, of all the placenta pathology features in this study, we observed that CHI and thrombi vasculopathy were the most common manifestations of placental pathology in SARS-CoV-2 infection. This finding was in accordance with other studies (48, 51, 64) reporting severe CHI composed of infiltrated immune cells such as CD68+ macrophages (Hofbauer cells), CD3+, CD8+ T lymphocytes, CD20+ B cells. These cells induce upregulation of pro-inflammatory cytokines such as IL-6, IP10, MIG; and this could be associated with poor fetal outcomes (32, 45, 49, 50). Taken together, SARS-CoV-2 infection on the placenta induces placental lesion, which is accompanied by massive infiltration of inflammatory cells, trophoblast necrosis, and fibrin deposition, and these are emerging hallmarks of SARS-CoV-2 infection in placenta.

## Limitations and strengths of the study

Our review has a few limitations. Studies reported are from different countries with different levels of pregnancies complication at a different gestational window were included in this study which may influence placental histological findings. Additionally, some studies were performed by subspecialists, which might affect the precision of the reported findings. Placenta pathological examination in some of the eligible studies was not available, making it difficult to conclude the placenta response to SARS-CoV-2 infection. Moving forward, the molecular and immunological methods employed by most of the publications included in this review is not without its detection limitation and precision (e.g., PCR, IHC, and ISH), making it difficult for accurate comparison. Lastly, the low sample size in most eligible studies also increased the probability of a high risk of bias in our study. Our study did not include the cause-effect of SARS-CoV-2 entry receptors expression on fetal-maternal samples on vertical transmission, and at the time of writing, there are no such existing literature. We report that the variability of these receptor expressions at different pregnancy window might support the plausibility of the vertical transmission in early gestation period. Overall, the assessment of the risks of bias in the studies reporting evidence of fetoplacental transmission was generally low (Supplementary Table 1), most were based on small sample sizes, strict access to samples stored in biobanks, and investigators being aware of SARS-CoV-2 positive samples before performing the experiments. We recommend that clinical studies including a large sample to be conducted because it would be helpful to provide guidelines for managing and preventing vertical transmission during the first trimester.

One of the major strengths of this study is that it documented and showed the plausibility of severe placental inflammation to SARS-CoV-2 infection. This is most likely to occur during the first trimester. Our study also finds that maternal-fetal transmission in COVID-19 positive pregnant women is rare, and the mechanism of SARS-CoV-2 fetoplacental transmission is still not clear.

## Conclusion

The main finding in our review is that there is no clear consensus on vertical transmission of SARS-CoV-2 during pregnancy, and the placenta is less susceptible to SARS-CoV-2 infection in the third trimester. Most of the studies in our review reported neonatal samples were negative for SARS-CoV-2 but showed the possibility of maternal antibodies in neonate samples such as amniotic fluid. The findings of this study increase our understanding of the possibility of fetoplacental transmission of SARS-CoV-2 in the general population. COVID-19 positive pregnant women in the early trimester should be carefully monitored in order to reduce severe complications for both mother and the fetus. It will also be interest to investigate whether early placental infection with SARS-CoV-2 is associated with fetal demise, rather than the systemic effect of maternal SARS-CoV-2 infection on developing fetus. Further studies on these topics are necessary to address questions on fetoplacental transmission of SARS-CoV-2 and to provide guidelines for the care and management of COVID-19 positive pregnant women.

## Data availability statement

The original contributions presented in this study are included in the article/Supplementary material, further inquiries can be directed to the corresponding author/s.

## Author contributions

HE: conceptualization, methodology, and data curation. HE, OA, JS, and MF: validation. HE, OA, JS, and MF: investigation. HE and OA: resources. HE, FE, and OA: analysis. HE, OA, JS, MF, and CD: writing—original draft preparation. HE, OA, JS, MF, CD, and FE: writing—review and editing. FE and OA: supervision. All authors read and agreed to the published version of the manuscript.
