# Corrigendum: Fetoplacental transmission and placental response to SARS-CoV-2: evidence from the literature

**DOI:** 10.3389/fmed.2023.1272249

**Published:** 2023-08-14

**Authors:** Henry C. Ezechukwu, Jiahua Shi, Muinah A. Fowora, Cornelius A. Diya, Faiz Elfaki, Oyelola A. Adegboye

**Affiliations:** ^1^Department of Medical Biochemistry, EKO University of Medicine and Health Sciences, Lagos, Nigeria; ^2^School of Human Sciences, University of Western Australia, Perth, WA, Australia; ^3^School of Medical, Indigenous and Health Sciences, University of Wollongong, Wollongong, NSW, Australia; ^4^Illawarra Health and Medical Research Institute, University of Wollongong, Wollongong, NSW, Australia; ^5^Department of Molecular Biology and Biotechnology, Nigerian Institute of Medical Research, Lagos, Nigeria; ^6^Department of Mathematics, Physics and Statistics, College of Arts and Sciences, Qatar University, Doha, Qatar; ^7^Public Health and Tropical Medicine, College of Public Health, Medical and Veterinary Sciences, James Cook University, Townsville, QLD, Australia; ^8^Australian Institute of Tropical Health and Medicine, James Cook University, Townsville, QLD, Australia

**Keywords:** SARS-CoV-2, placenta, vertical transmission, COVID-19, maternal-child

In the published article, there was an error in the correspondence section. **Faiz Elfaki** should appear as a corresponding author.

The authors apologize for this error and state that this does not change the scientific conclusions of the article in any way. The original article has been updated.

