# Supplementary material for: Fetoplacental transmission and placental response to SARS-CoV-2: Evidence from the literature
Source: Front Med (Lausanne). 2022 Aug 16;9:962937. doi: 10.3389/fmed.2022.962937 (PMC9426356; doi:10.3389/fmed.2022.962937)
Supplement: Supplementary file 1 [file Table_1.pdf]

Supplementary Table S1: Risk of bias assessment on the selected studies reporting evidence of possible fetoplacental transmission (n =15) . Low (-), high (+), unclear (or questionable) (?)

S/N	Selected studies	Randomization of samples (selection bias)	Homogeneity of the samples (selection bias)	Blinding of personnel while performing the experiment (performance bias)	Precision on the method of detection (detection bias)	Incomplete or omission on data outcome (attrition bias)	Selective reporting (reporting bias)
1	Fenizia et al (32)	-	-	?	-	+	-
2	Cui et al (33)	?	?	-	-	-	-
3	Li et al (35)	-	?	+	+	?	+
4	Lu et al (35)	-	?	+	?	-	-
5	Facchetti et al (37)	-	-	?	-	+	+
6	Faure-Bardon et al (27)	?	-	?	-	?	?
7	Valdespino-Vazquez et al (61)	?	-	?	-	-	-
8	Dong et al (8)	+	-	?	-	+	+
9	Santana et al (3)	?	-	?	-	-	-
10	Schwartz et al (51)	?	-	-	-	-	+
11	Shook et al (63)	-	-	?	-	-	-
12	Vivanti et al (68)	+	-	?	-	-	-
13	Alouini et al (65)	-	-	?	-	+	+
14	Sinaci et al (24)	-	-	?	-	-	-
15	Garrido-Pontnou et al (43)	?	-	?	-	-	-
